# Supplementary material for: Potential Modulation of Inflammation by Probiotic and Omega-3 Supplementation in Elderly with Chronic Low-Grade Inflammation—A Randomized, Placebo-Controlled Trial
Source: Nutrients. 2022 Sep 27;14(19):3998. doi: 10.3390/nu14193998 (PMC9573426; doi:10.3390/nu14193998)
Supplement: Supplementary file 1 [file nutrients-14-03998-s001.zip › nutrients-1795217-supplementary.pdf]

S1. Exclusion Criteria

1. Diagnosis of type 1 and/or type 2 diabetes
2. Current or within 4 weeks use of probiotic supplement prior to inclusion
3. More than 4 hours/week exercise habits
4. Immobile, defined as the inability to participate in all study related procedures
5. Dietary intake of fatty fish, fish oils containing omega-3 or pure omega- 3 supplements more than 2 times/week
6. History of complicated gastrointestinal surgery
7. Diagnosed Inflammatory Bowel Disease (IBD)
8. Current diagnosis of psychiatric disease/s or syndromes
9. Systemic use of antibiotics and/or steroid medication in the last 4 months prior to inclusion time
10. Use of any NSAID (Non-Steroidal Anti-Inflammatory Drugs) more than 3 times a week for the last 2 months and any time 3 days prior inclusion or at any time 3 days prior the barrier function test
11. Consumption of any NSAID up until 7 days prior to inclusion
12. Any condition which could interfere with the intestinal barrier function (e.g. gluten sensitivity, lactose intolerance, celiac disease, IBS, IBD) as decided by the principal investigators´ discretion
13. Drinking more than 9 standard cups of alcohol per week and/or more than 3 standard cups of alcohol per occasion
14. Regular smoking, use of snuff, nicotine or e-cigarette use
15. Regular use, for more than three times a week for the last 2 months and any time 7 days prior to inclusion, of medications which according to the principal investigator can have an anti-inflammatory effect or affect in any way the intestinal barrier function or have an impact on the study analysis (e.g. laxatives, anti-diarrheal, anti-cholinergic)
16. Any disorder which according to the principal investigator can have an anti-inflammatory effect and/or can affect the intestinal barrier function, or that can impact an adequate analysis of the study outcomes
17. After being included in the study, starting with a medication/treatment or medical intervention that could potentially influence the study participation and/or the study analysis (e.g. the event of a fracture)
18. Radical change in diet (e.g. becoming vegetarian or if they discover that they are lactose intolerant) during the study period
19. Allergic to fish
20. Allergic to milk- or soy protein

S2. Blood sample collection and analysis protocols

Blood collected in serum tubes was centrifuged for 15 min at 3000× g prior to analysis. hs-CRP levels in serum were analyzed using the CardioPhase hsCRP assay (REF 06837459, Siemens Healthcare, Germany) using the Siemens ADVIA 1800 Chemistry System (Siemens Healthcare) according to hospital routines at the laboratory of clinical chemistry, Örebro University Hospital, Örebro, Sweden. Glucose and insulin were similarly analyzed at the clinical chemistry in Örebro; glucose was analyzed by the ADVIA Chemistry Glucose Hexokinase_3 (GLUH_3) from venous blood collected in vacuum tubes containing citric acid, citrate and sodium fluoride, while insulin was analyzed using the ADVIA Centaur Insulin assay (a two-site sandwich immunoassay using direct chemiluminescent technology). Furthermore, Vitamin D levels were analyzed in serum according to clinical routines at Sindelfingen laboratory GbR, Sindelfingen, Stuttgart, Germany.

Cytokine concentrations in serum were quantified using the V-PLEX Proinflammatory Panel 1 Human Kit (MSD®; catalog #: K15049D-1) according to the manufacturer's protocol. Two-fold dilutions of samples and controls were applied according to manufacturer's recommendations. Plates were incubated at 750 rpm and were analyzed approximately three minutes after adding read buffer using the MSD QuickPlex Multiplex plate reader.

For analysis of L-PUFAs, blood collected in ethylenediaminetetraacetic acids (EDTA)-coated tubes was centrifuged at 500 g for 7.5 min at 15 °C, followed by plasma separation and storage at −80 °C. Prior to analysis all samples were shipped, on dry ice, to the Institute of Clinical Nutrition, University of Hohenheim, Stuttgart, Germany, where the analysis was performed as follows: 100 µL plasma, 2 µL 10,11-dichloro-undecanoic acid (11:0) as internal standard and 2 mL methanol (Carl Roth GmbH, Karlsruhe, Germany) with 1% sulphuric acid for transesterification were added. The following unsaturated fatty acids were measured in: monounsaturated fatty acids (PUFAs): 14:1 n-5 (myristoleic acid), 16:1, 16:1 n-7 (palmitoleic acid), 17:1, 18:1 n-9 c (oleic acid), 18:1 (isomer of oleic acid), 20:1 n-9 (gondoic acid) and polyunsaturated fatty acids (PUFAs), the following were measured: 18:2 n-6 (LA), 20:2 n-6 (eicosadienoic acid), 18:3 n-6 (γ-linolenic acid), 18:3 n-3 (ALA), 20:3 n-6 (dihomogammalinolenic acid), 20:4 n-6 (AA), 20:5 n-3 (EPA), 22:5 n-3 (DPA), 22:6 n-3 (DHA). To measure the n-6/n-3 ratio, plasma levels of total n-6 (18:2 n-6, 20:2 n-6, 18:3 n-6, 20:3 n-6, 20:4 n-6) and total n-3 (18:3 n-3, 20:5 n-3, 22:5 n-3, 22:6 n-3) were measured and divided. For the AA/EPA ratio the fatty acids were divided through each other. The method has been previously described in a previous study[28].

Finally, Plasma I-FABP concentrations in Li-heparin plasma (BD Vacutainer® LH PST™ II (BD), 8-10 inversions, centrifugation within 20 min, 1500 g, 10 min, 4°C, brakes not activated) were measured using an ELISA (HK406, HycultBiotech, Uden, The Netherlands) following the manufacturer's instructions. The detection range of this assay was specified to be 47 to 3,000 pg/ml.

S3. Chocolate bar ingredients

ONEWAY CHOCOLATE CHIP BAR

Ingredients: Protein blend (whey protein isolate, milk protein [milk]), consistency agent (sorbitol), sugar-free chocolate (vegetable fat [palm kernel oil, palm], dietary fiber [inulin], fat-reduced cocoa powder [20%], milk protein concentrate [milk medium, malt powder soy lecithin), flavor), consistency agent (glycerol), sunflower oil, sugar-free chocolate coating

(vegetable fat [palm kernel oil, palm], dietary fiber [inulin], fat-reduced cocoa powder [20%], milk protein concentrate [milk], sweetener [maltitin]), flavor), sweetener (maltitol), collagen hydrolyzate, oligofructose, water, cocoa nibs (3%), emulsifier (soy lecithin), flavor, salt.

Nutrition declaration per bar (55 g) per 100 gEnergy947 kJ / 231 kcal1722 kJ / 420 kcalFat9,9 g18 g- of which saturated fat4,8 g8,8 gCarbohydrates13,7 g25 g- of which sugars * 0.4 g0,7 g- of which lactose0, 1 g0.2 g- of which starch0.06 g0.1 g- of which sucrose0.2 g0.4 g- of which polyols13.2 g24 g- of which dietary fiber4.9 g9 gProtein19.3 g35 gSalt20 mg37 mg * Contains naturally occurring sugars

S4. Short-chain fatty-acid sample preparation and analysis

After collection, samples were immediately put into a pre-frozen cooling transport systems (Sarstedt, Germany) and stored -20°C until delivered to the study unit where samples were stored at −80°C. Prior to analysis all samples were shipped, on dry ice, to the Institute of Clinical Nutrition, University of Hohenheim, Stuttgart, Germany, where the analysis was performed as follows: raw fecal samples were homogenized, weighed (ca. 400 mg), diluted 1:4 with distilled water and 100 μL 50% phosphoric acid (Carl Roth GmbH, Karlsruhe, Germany) were added. Samples were homogenized with a whirlmix and centrifuged (20,000g at 4 °C, 20 min) twice (5417R, Eppendorf, Hamburg, Germany). The supernatant was drawn up and filtered with a syringe filter with glass fiber (WIC 79545, Wicom, Heppenheim, Germany) to an autosampler glas (WIC 42100 with crimp caps, Wicom) with Micro Inserts (No 548-00060, VWR International GmbH, Darmstadt, Germany). With a capillary gas chromatograph (Clarus 690, Perkin-Elmer, Waltham, MA, USA), using a liquid autosampler with a capillary column (Cat. # N9316354, Perkin Elmer) with standards (Volatile Free fatty acid Mix CRM46975, Merck Schuchhardt OHG, Hohenbrunn, Germany), 1L filtrate was analyzed. For data integration, the software total-Chrome Version 6.3.4 (Perkin-Elmer, Waltham, MA, USA) was used. For fecal dry mass quantification, 200 mg fecal samples were weighed and dried for 12 h at 103°C. SCFA data are expressed in relation to dry mass and identified by comparing the retention times of the respective peaks in the sample and standard chromatograms.

S5. Mobility measurements

Out of the 76 participants, 2 participants were unable to complete the SST test and were excluded from analysis. There was no statistically significant change in time (seconds) to complete the five- repetition sit-to-stand test between the groups, baseline corrected values for placebo vs treatment group were -0.74(1.84) and -0.12(2.37) respectively. Both groups improved slightly, but non-significant, after the intervention.

Nor did the intervention infer any significant improvements in activity levels as assessed with the WOMAC questionnaire; baseline corrected values (points) for placebo vs treatment group were -3.08(12.5) and -1.05(9.90) respectively.
